# Molecular characterization and transmission pattern of tetracycline resistance determinants in tigecycline and carbapenem resistant Klebsiella pneumoniae isolates at a tertiary care hospital in India

**DOI:** 10.1099/acmi.0.001017.v4

**Published:** 2026-03-12

**Authors:** Jyoti Chaudhary, Richa Sinha, Irfan Hasan, Ranjeet Singh Chauhan, Chinmoy Sahu

**Affiliations:** 1 Department of Microbiology, Sanjay Gandhi Postgraduate Institute of Medical Sciences, C Block, 2nd Floor, SGPGIMS Lucknow, UP – 226014, India; 2 Department of Microbiology, Dr. Ram Manohar Lohia Institute of Medical Science, Ground Floor, RML Lucknow, UP – 226010, India; 3 Department of Clinical Immunology and Rheumatology, Sanjay Gandhi Postgraduate Institute of Medical Science, C Block, Ground Floor, SGPGIMS Lucknow, UP – 226014, India

**Keywords:** carbapenem resistance, *Klebsiella pneumoniae*, tigecycline resistance

## Abstract

**Background.** The increasing prevalence of tigecycline and carbapenem-resistant *Klebsiella pneumoniae* (CRKP) poses a serious challenge, especially in resource-limited settings. Its ability to exchange resistance genes with other bacteria accelerates the spread of multidrug resistance. While carbapenems and tetracyclines have been used effectively against *K. pneumoniae*, resistance to these agents is now rising globally, narrowing available treatment options.

**Objective.** The study aimed to determine the phenotypic and genotypic prevalence of carbapenem and tetracycline resistance in *K. pneumoniae* isolates along with the transferability pattern of carbapenem and tetracycline resistance genes in these isolates.

**Methodology.** Clinical isolates from pus and respiratory samples were identified using biochemical tests and MALDI-TOF MS. Antimicrobial susceptibility test was performed by the Kirby–Bauer disc diffusion method, and MICs were determined by the broth microdilution test method. PCR was performed to detect carbapenemase (*bla*_NDM_, *bla*_OXA-48_ and *bla*_KPC_) and tetracycline resistance genes [*tet(A)*, *tet(B)*, *tet(K)*, *tet(M)* and *tet(S)*], followed by Sanger sequencing for validation. Conjugation assays assessed gene transferability.

**Results.** Out of 152 CRKP isolates, 20.4% (31 out of 152) were found to be resistant to tigecycline. All tigecycline-resistant isolates exhibited complete resistance (31 out of 31; 100%) to ceftazidime, ciprofloxacin and omadacycline. Additionally, resistance to amikacin and cefoperazone-sulbactam was observed in 87.1% (27 out of 31) and 77.4% (24 out of 31) of the isolates. Resistance to minocycline and colistin was detected in 51.6% (16 out of 31) and 29.0% (9 out of 31) of the isolates, respectively.

PCR analysis revealed that 51.6% (16 out of 31) of the isolates carried the *bla*_OXA-48_ gene, and 29.0% (9 out of 31) carried the *bla*_NDM_ gene. None of the isolates harboured the *bla*_KPC_ gene. With respect to tetracycline resistance determinants, the *tet(A)* gene was detected in 12.9% (4 out of 31) of the isolates, and the *tet(B*) gene in 3.2% (1 out of 31), while *tet(K)*, *tet(M)*, *tet(S)* and *bla*_KPC_ were not detected in any isolate.

Conjugation assays demonstrated that plasmids carrying *bla*_NDM_ and *bla*_OXA-48_ were transferable to a recipient strain, indicating their potential for horizontal gene transfer. In contrast, plasmids harbouring *tet(A)* and *tet(B)* genes were not transferable under the experimental conditions.

**Conclusion.** Tigecycline-resistant *K. pneumoniae* isolates showed high multidrug resistance, with transferable *bla*_NDM_ and *bla*_OXA-48_ genes. In contrast, chromosome and plasmid-borne tetracycline resistance genes *tet(A)* and *tet(B)* were non-transferable, indicating limited horizontal spread.

## Data Summary

The data sets indicated in this study can be found in online repositories. The nucleotide sequences of the *tet(A)* and *tet(B)* genes were submitted in the NCBI GenBank database under accession nos. PQ432290, PQ432289, PV091799, PV091800 and PV091803, respectively.

## Introduction

Antimicrobial-resistant *Klebsiella pneumoniae* has emerged as a significant challenge in healthcare settings worldwide, particularly due to its increasing resistance to last-resort antibiotics such as carbapenems and tigecycline [1]. *K. pneumoniae* is a key opportunistic pathogen responsible for various nosocomial infections, including pneumonia, urinary tract infections, sepsis and wound infections, especially in immunocompromised or critically ill patients [2,3]. Reported mortality rates are high in hospitalized patients infected with *K. pneumoniae*, with pooled estimates of 17% at 7 days, 24% at 14 days, 29% at 30 days, 34% at 90 days and 29% for overall in-hospital deaths [4].

Carbapenem-resistant *K. pneumoniae* (CRKP) is classified by the World Health Organization as a critical-priority pathogen because of its high mortality rates, multidrug resistance and ability to spread rapidly in hospital environments [5]. Infections caused by multidrug-resistant *K. pneumoniae* are typically treated with last-resort antibiotics such as carbapenems and tigecycline. However, the increasing resistance of this pathogen to both drugs has significantly limited effective treatment options [6,8].

Tetracycline resistance in *K. pneumoniae* is predominantly mediated by *tet* genes, such as *tet(A)* and *tet(B)*, which encode efflux pumps that reduce intracellular drug concentrations. These genes are often located on mobile genetic elements like plasmids, which facilitate horizontal gene transfer across bacterial species [9,11]. The co-existence of tetracycline- and carbapenem-resistant genes on conjugative plasmids raises concern about the potential for their simultaneous dissemination, further complicating treatment and infection control strategies [12,13].

Several studies have reported the prevalence of carbapenemase genes, such as *bla*_NDM_ and *bla*_OXA-48_, in *Enterobacteriaceae*, including CRKP isolates; however, data on the prevalence of tetracycline resistance genes remain limited [14,16]. Moreover, there is currently no information available on the genetic location and mobility of *tet* genes in clinical isolates from India. The presence of these resistance determinants on plasmid, along with their potential for conjugative transfer, highlights the need for detailed molecular characterization to understand their role in the dissemination of antimicrobial resistance [10].

In the present study, we investigated *K. pneumoniae* isolates from a tertiary care hospital in North India that were co-resistant to carbapenems and tigecycline. We characterized the antimicrobial resistance profiles, screened for key resistance genes and analysed their plasmid association and transferability through conjugation assays. This study aims to provide new insights into the genetic basis and dissemination of carbapenem and tetracycline resistance in CRKP, with a focus on the tetracycline resistance gene, contributing to the broader understanding of multidrug resistance in clinical settings. To our knowledge, this is the first investigation from India that explores both the genomic localization and mobility of these carbapenem and tetracycline resistance determinants in CRKP.

## Objectives of the study

This study aimed to assess the occurrence and genetic basis of tigecycline resistance in CRKP isolates. It also sought to identify the chromosomal or plasmid localization of carbapenemase and tetracycline resistance genes, along with evaluating their ability to transfer between isolates.

- To determine the prevalence, antibiotic susceptibility profile and MIC in tigecycline-resistant *K. pneumoniae* (TRKP) among carbapenem-resistant isolates.
- To determine the genetic characterization of carbapenem resistance (*bla*_NDM_, *bla*_KPC_ and *bla*_OXA-48_) and tetracycline resistance gene [(*tet(A)*, *tet(B)*, *tet(K)*, *tet(M)* and *tet(S)*] among these isolates.
- To determine the transferability pattern of carbapenemase and tetracycline resistance genes in *K. pneumoniae* clinical isolates.

## Methods

This observational, cross-sectional and descriptive study was conducted at the Sanjay Gandhi Postgraduate Institute of Medical Sciences, Lucknow, India. Clinical specimens were collected from various hospital wards and processed in the Department of Microbiology. A total of 31 non-duplicate CRKP and TRKP isolates were obtained from pus and respiratory samples of hospitalized patients, presenting with a range of infections.

### Sample collection

This study included *K. pneumoniae* strains, isolated from various clinical specimens, such as pus and respiratory samples. The samples were collected as part of routine clinical procedures performed by attending physicians for patient care, without any direct interaction or intervention by the research team. Following collection, specimens were placed in sterile, leak-proof containers and promptly transported to the Microbiology Department for further analysis. To ensure patient confidentiality, all samples were anonymized prior to processing.

#### Pus sample

The affected area was irrigated with sterile normal saline to eliminate surface contaminants such as slough, necrotic debris and dried exudates. Pus and wound exudates were collected either by inserting a sterile cotton swab into the advancing edge of the wound or by aspiration using a sterile syringe. Collected specimens were promptly transferred to the laboratory in transport medium to preserve microbial viability during transit.

#### Endotracheal and transtracheal aspirate

Endotracheal (ET) aspiration involves aspirating secretions from the trachea using an ET tube, allowing for direct sampling of the lower respiratory tract for the detection and identification of micro-organisms causing respiratory tract infection. A suction catheter was placed through the ET tube, and then, suction was used while the catheter was withdrawn. Saline lavage was administered as needed to help remove secretions.

#### Sputum sample

Sputum samples were collected in the early morning from conscious patients presenting with lower respiratory tract infections. To reduce contamination from upper respiratory flora, patients were advised to rinse their mouths with water prior to sample collection. They were then instructed to produce a deep cough to expectorate sputum into a sterile, wide-mouthed container with a secure lid. Only dense, mucopurulent secretions originating from the lower respiratory tract were deemed acceptable for diagnostic evaluation.

### Sample processing and preservation

For bacterial isolation, all collected specimens were inoculated onto blood agar and MacConkey agar plates and incubated aerobically at 37 °C for 24 h. Preliminary identification of bacterial isolates was performed using the standard conventional biochemical tests and further validated by an automated MALDI-TOF MS system (bioMérieux Inc., France) by using freshly grown overnight colonies on blood agar (Direct Colony Method). All non-repeated *K. pneumoniae* isolates were stored in 20% glycerol-supplemented Luria–Bertani medium at −40°C.

### Inclusion and exclusion criteria

Only Gram-negative bacterial isolates from pus and respiratory samples, isolated from patients with an age range of 18–60 years, were included in this study. This selection was based on the therapeutic use of tigecycline, which is commonly prescribed for managing infections of the respiratory tract, intra-abdominal region and skin and soft tissue infections. Isolates from other sample types like blood and urine were excluded in this study.

### Demographic data and statistical analysis

All the data were analysed by SPSS software (version 25), and proportions were compared using the chi-square test to determine the significant differences. Data were presented as percentage, frequency and specificity for categorical variables.

### Antimicrobial susceptibility test

An antimicrobial sensitivity testing of all collected isolates was done using the E-test method for tigecycline and colistin, and the Kirby–Bauer disc diffusion method for imipenem (10 µg), meropenem (30 µg), amikacin (30 µg), ciprofloxacin (5 µg), ceftazidime (30 µg), cefoperazone+sulbactam (75/30 µg) and minocycline (30 µg).

### Genetic screening of resistance gene

The DNA isolation of all *K. pneumoniae* isolates was done by the Qiagen mini prep kit. The conventional PCR was performed to detect the carbapenemase (*bla*_KPC_, *bla*_NDM_ and *bla*_OXA−48_) and tetracycline-resistant encoding genes {*tet(A)*, *tet(B)*, *tet(K)*, *tet(M*) and *tet(S)*}, using gene-specific primers and specific reaction conditions [17,19]. All PCR reactions were performed in 12.5 µl volumes containing 6.5 µl of 2X DreamTaq PCR Master Mix (Thermo Fisher, Carlsbad, USA), 1 µl of each primer (10 pmol µl^−1^), 2 µl nuclease-free water and 2 µl of isolated DNA (50 ng µl^−1^).

The used primer sequences, annealing temperatures and the product size are listed in Table S1, available in the online Supplementary Material. Amplified PCR products were analysed by electrophoresis in 1.5% agarose gel (containing ethidium bromide) at 75 V for 50 min in Tris-acetate EDTA buffer and visualized under a UV trans-illuminator.

*K. pneumoniae* isolates (CRkp21, CRkp22) from our previous studies were used as positive controls for carbapenemase genes, and *Escherichia coli* DH5α served as the negative control [18]. However, due to the unavailability of a confirmed positive control for tetracycline resistance genes in our laboratory, all visible PCR amplicons were subjected to Sanger sequencing to validate the presence of tetracycline resistance determinants.

### Sanger sequencing

The purified PCR product amplicons of tetracycline resistance gene were eluted from 1.5% agarose gel and subsequently sequenced by 3500 Series Genetic Analyzers (Thermo Fisher Scientific-IN). The obtained sequences were matched with the International Nucleotide Sequence Database Collaboration and further submitted on the NCBI portal by Bankit to retrieve the accession number.

### Broth microdilution test

The MIC determination by broth microdilution test assay was used to measure the *in vitro* activity of an antimicrobial agent against a *K. pneumoniae* isolate. The MICs of imipenem, meropenem, ceftazidime, ceftriaxone, ciprofloxacin, aztreonam, gentamicin, minocycline, omadacycline and colistin were determined using cation-adjusted Mueller–Hinton broth in 96-well microtiter plates according to the Clinical and Laboratory Standards Institute (CLSI) guidelines and for tigecycline using European Committee Antimicrobial Susceptibility Testing (EUCAST) guidelines [20,21]. Breakpoints for omadacycline in *K. pneumoniae* were not available in CLSI or EUCAST guidelines.

### Plasmid DNA isolation and PCR-based localization of resistance gene determinants

Plasmid DNA was extracted from all *K. pneumoniae* isolates that tested positive for either carbapenemase or tetracycline resistance genes using the Genetic Mini Prep Kit (NP-37105), in accordance with the manufacturer’s instructions. The concentration of the extracted plasmid DNA was quantified using a Nano Drop spectrophotometer, and DNA integrity was assessed by agarose gel electrophoresis.

To investigate whether the detected resistance genes were plasmid-borne, PCR amplification was performed using the plasmid DNA as a template. The PCR conditions were consistent with those used in the initial genetic screening of resistance genes. Successful amplification of the target genes from the plasmid DNA confirmed their plasmid localization, indicating their potential role in the horizontal dissemination of antimicrobial resistance among the *K. pneumoniae* isolates.

### Conjugation assay

To investigate the potential for horizontal gene transfer of carbapenemase and tetracycline resistance genes, conjugation experiments were carried out. Sodium azide-resistant *E. coli* J53 was used as the recipient strain. Transconjugants (Tc) were selected on MacConkey agar plates supplemented with sodium azide (100 µg ml^−1^), ampicillin (50 µg ml^−1^) and tigecycline (0.5 µg ml^−1^) [22]. Transconjugant colonies (*E. coli* J53) were confirmed through MALDI-TOF MS followed by an antibiotic susceptibility test and PCR amplification to verify the presence of the respective resistance genes.

## Results

### Collection, isolation and identification of *K. pneumoniae* isolates

In this prospective study, a total of 152 CRKP isolates were included. Among these, 20.4% (31 out of 152) exhibited resistance to tigecycline. The most commonly collected clinical specimens were respiratory samples, followed by pus samples, but the highest proportion of CRKP and TRKP isolates was observed in pus (54.83%), followed by transtracheal (TT) aspirates (32.25%), ET aspirates (6.45%) and sputum (6.45%). All specimens were submitted to the Department of Microbiology for diagnostic evaluation of hospitalized patients (Table 1).

**Table 1.** Specimen-wise distribution of *K. pneumoniae* isolates (*N*=31)

Sample type	Carbapenem resistance (%)	Tigecycline resistance (%)
Pus	52/152 (34.21%)	17/31 (54.83%)
TT aspirate	38/152 (25%)	10/31 (32.25%)
ET aspirate	23/152 (15.13%)	2/31 (6.45%)
Sputum	39/152 (25.65%)	2/31 (6.45%)

**Fig. 1.**
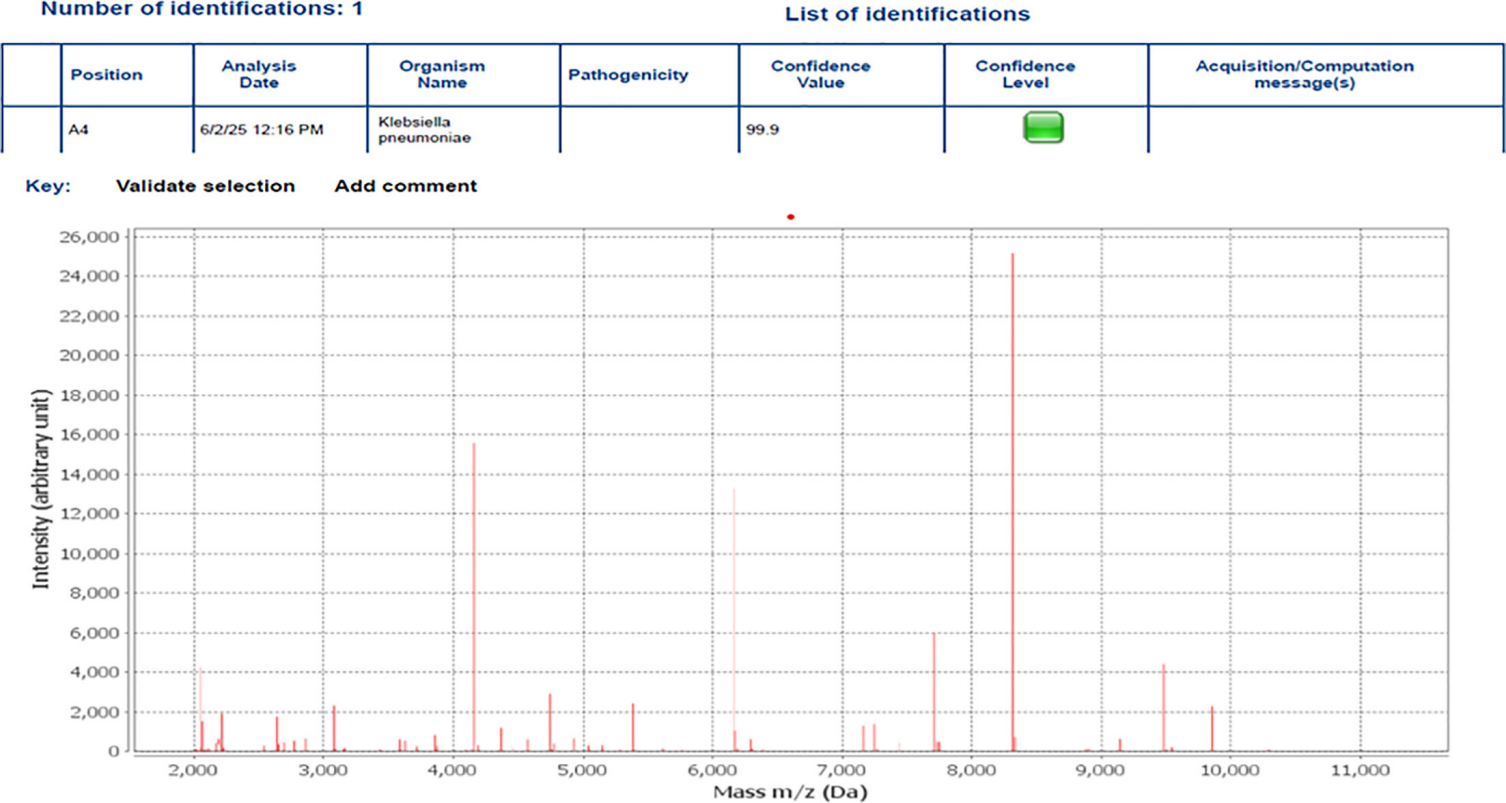
Identification of *K. pneumoniae* isolate (Kp1) by MALDI-TOF MS.

All isolates were Gram-negative, non-motile, rod-shaped, exhibiting typical colony morphology after 24 h of incubation. On blood agar, colonies appeared smooth, convex, 0.5–2 mm in diameter, translucent to opaque with entire margins. On MacConkey agar, the isolates produced large, pink, mucoid colonies due to lactose fermentation. Biochemical characterization revealed that all isolates were positive for catalase, citrate utilization, Voges-Proskauer (VP), urease and glucose fermentation. They tested negative for oxidase, methyl red (MR), indole, hydrogen sulphide (H₂S) production and phenylalanine deaminase (PDA) (Table 2). Final identification of all isolates of *K. pneumoniae* was confirmed using MALDI-TOF MS (Fig. 1).

**Table 2.** Biochemical test used to identify *K. pneumoniae* isolates

Biochemical test	Result (*K. pneumoniae*)
Catalase, OF glucose, citrate utilization, VP, urease	Positive
Oxidase, MR, indole, H_2_S production, PDA	Negative

### Demographic data of patients

Clinical and demographic analysis showed that the highest rate of *K. pneumoniae* isolation was from the critical care medicine (35.48%) followed by surgical gastroenterology (25.81%), nephrology (9.67%), anaesthesia (9.67%) and apex trauma centre (9.67%), while the lowest rate was from the neurosurgery (6.45%) and emergency medicine (3.22%) wards of hospitalized patients (Table 3). Among patients infected with TRAB, 83.87% (26 out of 31) were male, and 5 (16.13%) were female within the age range of 18–60 years (Table 4).

**Table 3.** Ward-wise distribution of *K. pneumoniae* isolates (*N*=31)

Hospital wards	No. of samples (%)
Critical care medicine	11 (35.48%)
Surgical gastroenterology	8 (25.81%)
Nephrology	3 (9.67%)
Anaesthesia	3 (9.67%)
Apex trauma centre	3 (9.67%)
Neurosurgery	2 (6.45%)
Emergency	1 (3.22%)

**Table 4.** Age- and gender-wise distribution of patients infected with *K. pneumoniae* isolates

Sex	Percentage	Age (years)
Male	26 (83.87%)	18–60
Female	5 (16.13%)	18–60

### Antimicrobial susceptibility profile and MIC

Antibiotic susceptibility testing of 152 *K*. *pneumoniae* isolates was conducted using the Kirby–Bauer disc diffusion method. Among these, 20.4% (31 out of 152) were found to be co-resistant to both tigecycline and carbapenems. These multidrug-resistant isolates showed complete resistance (100%) to ceftazidime and ciprofloxacin. High resistance rates were also observed for amikacin and cefoperazone-sulbactam (SCF) in 87.1% (27 out of 31) and 77.42% (24 out of 31) of the isolates, respectively. In contrast, lower resistance frequencies were noted for minocycline (51.61%, 16 out of 31) and colistin (29.04%, 9 out of 31), suggesting partial retention of susceptibility to these agents (Table 5).

**Table 5.** Antibiotic resistance profile of *K. pneumoniae* by Kirby–Bauer disc diffusion method (*N*=31)

Antibiotic groups	Name of antibiotics	Symbols of disc	Susceptible (%)	Resistant (%)
Carbapenems	Imipenem	IMP (10 µg)	0% (0/31)	100% (31/31)
	Meropenem	MEM (10 µg)	0% (0/31)	100% (31/31)
Aminoglycosides	Amikacin	AKN (30 µg)	12.9% (4/31)	87.1% (27/31)
Fluoroquinolones	Ciprofloxacin	CIP (5 µg)	0% (0/31)	100% (31/31)
Combination (BL+BLI)	Cefoperazone+sulbactam	SCF (75/30 µg)	22.58% (7/31)	77.42% (24/31)
Tetracycline	Minocycline	MNO (30 µg)	48.38% (15/31)	51.61% (16/31)
	Tigecycline	Tg (E-strip)	0% (0/31)	100% (31/31)
Cephalosporin	Ceftazidime	CAZ (30 µg)	0% (0/31)	100% (31/31)
Polymyxin	Colistin	CT (E-strip)	70.96% (22/31)	29.04% (9/31)

### Genetic screening and Sanger sequencing

Single-plex conventional PCR assays were utilized to detect the presence of carbapenemase and tetracycline resistance genes, specifically *bla*_NDM_, *bla*_OXA-48_, *bla*_KPC_, *tet(A)*, *tet(B)*, *tet(K)*, *tet(M*) and *tet(S)*. Among the 31 *K*. *pneumoniae* isolates analysed, the most frequently identified carbapenem resistance gene was *bla*_OXA-48_-like, found in 51.6% (16 out of 31) of isolates, followed by *bla*_NDM_, present in 29% (9 out of 31) isolates. Notably, none of the isolates tested positive for the *bla*_KPC_ gene.

**Fig. 2.**
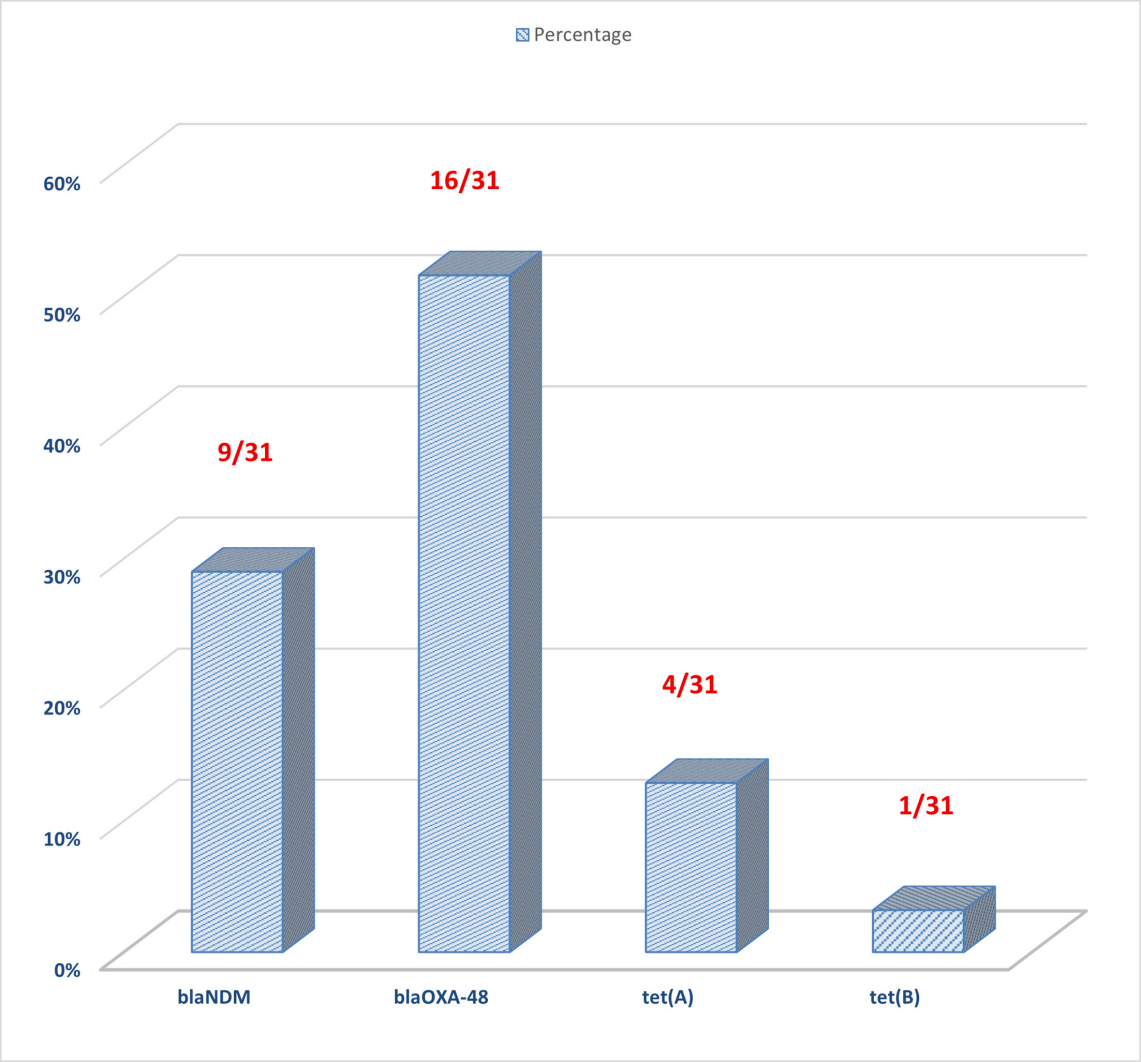
Genotypic prevalence of *bla*_NDM_, *bla*_OXA-48_, *tet*(*A)* and *tet(B)* genes.

Regarding tetracycline resistance determinants, *tet(A)* was detected in 12.9% (4 out of 31) of the isolates, and *tet(B*) was observed in 1 isolate (3.2%; 1 out of 31). The genes *tet(K)*, *tet(M)* and *tet(S)* were not detected in any of the tested isolates (Fig. 2). These results indicate a selective distribution of resistance genes within the study population, with a higher prevalence of carbapenemase genes compared to tetracycline resistance genes.

Among the isolates harbouring the *tet(A)* gene, three (Kp1, Kp4 and Kp20) were found to co-harbour carbapenemase genes. Specifically, Kp1 and Kp4 carried both *bla*_NDM_ and *bla*_OXA-48_-like genes, whereas Kp20 was positive for *bla*_OXA-48_-like alone. The remaining *tet(A)-*positive isolate (Kp30) did not carry any of the screened carbapenemase genes. Additionally, one isolate (Kp20) possessing *tet(A)* also concurrently harboured the *tet(B)* gene, indicating the co-existence of multiple tetracycline resistance determinants within a single strain. Isolates containing *tet(A) and tet(B)* genes were Kp1, Kp4, Kp20, Kp30 and Kp20, respectively, that were sequenced for further validation and submitted by Bankit on NCBI portal. All the accession numbers were mentioned in the data availability section.

### Minimum inhibitory concentration

The MICs were determined using the broth microdilution method. The MIC results demonstrated that the majority of isolates exhibited a high degree of resistance to the tested antimicrobial agents, with the exception of minocycline and colistin (Table 6). In *K. pneumoniae* isolates harbouring the tetracycline resistance gene, the MIC of tigecycline was increased ninefold in one isolate and eightfold in the remaining three isolates, relative to the established clinical susceptibility breakpoint (0.5 µg ml^−1^) (Table 7). Additionally, MIC values for imipenem and meropenem in some isolates increased substantially, reaching up to ≥256 µg ml^−1^ in certain isolates, as presented in Table 7.

**Table 6.** MIC of *K. pneumoniae* by broth microdilution method (*N*=31)

Organism/no. of isolates	Antibiotics (range tested in µg/ml)	CLSI/EUCAST breakpoint ≤S/≥R		No. of isolates with MIC (µg/ml)								% R
			≤1	≤2	4	8	16	32	64	128	≥256	
*K. pneumoniae* (31)	Tigecycline*	≤1/≥2	–	2	4	6	**8**	4	**5**	2	–	100
	Minocycline (0.25–0.25–256)	≤4/≥16	–	–	–	–	1	4	3	2	6	51.6
	Omadacycline (0.25–256)	na	–		5	6	**8**	3	4	**2**	3	100
	Imipenem (0.25–256)	≤1/≥4	–	–	1	2	1	3	2	4	**≥18**	100
	Meropenem (0.25–256)	≤1/≥4	–	–	–	4	2	3	3	2	≥17	100
	Ceftazidime (1–512)	≤4/≥16	–	–	–	–	–	–	–	–	31	100
	Ceftriaxone (1–512)	≤1/≥4	–	–	–	–	–	–	–	–	31	100
	Aztreonam (1–512)	≤4/≥16	–	–	–	–	–	–	–	–	31	100
	Gentamicin (1–512)	≤4/≥16	–	–	–	–	–	–	–	–	31	100
	Ciprofloxacin (1–512)	≤1/≥4	–	–	–	–	–	–	–	–	31	100
	Colistin* (1–512)	≤2/>2		–	–	–	2	3	2	1	1	29.1

*EUCAST Guidelines.

**Table 7.** MIC of *tet(A)* and *tet(B)* containing *K. pneumoniae* (*N*=4)

Isolate ID	MIC		
	Tigecycline	Imipenem	Meropenem
Kp1	64	˃256	˃256
Kp4	64	˃256	˃256
Kp20	128	128	64
Kp30	64	32	16

### Plasmid localization

Plasmid DNA was extracted from isolates Kp1, Kp4, Kp20 and Kp30 and subjected to PCR to evaluate the presence of genes on plasmids. Most of the isolates (Kp1, Kp4 and Kp20) showing positive PCR results for the carbapenemase genes (*bla*_NDM_ and *bla*_OXA-48_) showed successful amplification from plasmid DNA, similar to amplification from total genomic DNA, indicating their association with plasmids. However, among the four *tet(A)*-positive isolates, only two (Kp1 and Kp4) demonstrated amplification from plasmid DNA, suggesting variability in the genetic location of this gene, while two *tet(A)-*positive isolates (Kp30, Kp20) and one *tet(B)-*positive isolate (Kp20) showed negative PCR result from plasmid DNA (Table 8). Isolates confirmed to carry *bla*_NDM_, *bla*_OXA-48_ and *tet(A)* genes on plasmids were further assessed for their potential to mediate horizontal gene transfer. These analyses revealed that *bla_NDM_* and *bla_OXA-48_* were exclusively plasmid-borne, while *tet(A)* may be located on both plasmids and the chromosome in different isolates.

**Table 8.** Comparative analysis of gene presence on genomic DNA versus plasmid DNA in tetracycline gene-containing isolates

Selected isolates	Genomic DNA PCR amplification	Plasmid DNA PCR amplification
Kp1	*bla*_NDM_, *bla*_OXA-48_, *tet(A)*	*bla*_NDM_, *bla*_OXA-48_, *tet(A)*
Kp4	*bla*_NDM_, *bla*_OXA-48_, *tet(A)*	*bla*_NDM_, *bla*_OXA-48_, *tet(A)*
Kp20	*bla*_OXA-48_, *tet(A), tet(B)*	*bla*_OXA-48_
Kp30	*tet(A)*	

### Horizontal transfer potential of resistance genes

Conjugation experiments were conducted in four isolates (Kp1, Kp4, Kp20 and Kp30) to investigate the potential for plasmid-mediated transfer of resistance determinants. The *bla*_NDM_ genes from isolates Kp1 and Kp4, and the *bla*_OXA-48_-like gene from isolate Kp20, were successfully transferred to *E. coli* J53 recipient strains, while the *bla*_OXA-48_-like gene from isolates Kp1 and Kp4 was non-transferable. All three donor isolates also harboured a *tet(A)* gene. The presence of transferred carbapenemase genes in the resulting transconjugants (Tc-Kp1, Tc-Kp4 and Tc-Kp20) was verified by PCR targeting plasmid DNA.

Antimicrobial susceptibility testing of the transconjugants showed that Tc-Kp1 and Tc-Kp20 were resistant to both imipenem and meropenem. Tc-Kp4 demonstrated resistance to imipenem but remained susceptible to meropenem.

Interestingly, the *tet(A)* gene, although located on plasmid DNA in isolates Kp1 and Kp4, was not transferred to the recipient strain under the experimental conditions. This observation suggests that the *tet(A)* gene may reside on non-conjugative plasmids or be subject to transfer limitations, unlike the carbapenem resistance genes, which showed efficient plasmid-mediated mobilization (Table 9).

**Table 9.** Transferability of carbapenem and tetracycline resistance genes via conjugation assays

Gene	Selected isolates	AST profile of wild-type		Transconjugants (Tc)	AST profile of transconjugants	
		Imipenem	Meropenem		Imipenem	Meropenem
*bla*_NDM_	Kp1	R	R	Tc-Kp1	R	R
	Kp4	R	R	Tc-Kp4	R	S
*bla*_OXA-48_	Kp1	R	R	nt		
	Kp4	R	R	nt		
	Kp20	R	R	Tc-Kp20	R	R
*tet(A)*	Kp1	R	R	nt		
	Kp4	R	R	nt		

## Discussion

The widespread use of antibiotics in clinical practice, agriculture and livestock has significantly contributed to bacterial antimicrobial resistance. This resistance includes transmissible tigecycline resistance, which undermines the efficacy of tigecycline in treating infections caused by carbapenem-resistant *Enterobacteriaceae* [23]. In contrast to worldwide data, Indian studies indicate that tigecycline resistance is more common in *K. pneumoniae* and *Acinetobacter baumannii* when compared to any other Gram-negative bacterial species [24,25]. Although limited studies from India have examined tigecycline resistance among bacterial isolates, no research to date has explored the genetic mechanisms underlying tigecycline resistance [26]. Therefore, we aimed to investigate the prevalence of tigecycline resistance in clinical isolates of *K. pneumoniae* and performed genetic characterization to better understand the transferability patterns of resistance in this pathogen.

*K. pneumoniae* is an emerging threat due to its resistance to most last-line antibiotics, posing significant challenges, especially in hospital settings. Economically developed areas, such as China, with advanced medical systems and higher antibiotic exposure, alongside high population density, contribute to increased isolation rates of the bacteria. Despite a rising trend in isolation rates, resistance rates have shown a decreasing trend over recent years [27].

*K. pneumoniae* exhibits resistance in China due to specific genes, with the most prevalent being CTX-M-1 (41.9%), SHV-11 (41.8%), TEM (39.5%), CTX-M-15 (35.3%), *bla*_KPC-2_ (14.6%) and *bla*_NDM-1_ (6.7%), while in India, *bla*_OXA_ and *bla*_NDM_ are the main cause of genetic resistance in this isolate [28]. A study from our centre reported a high prevalence of *bla*_NDM_ (50%) previously [29]. Another multicentric study from India reported the presence of *bla*_OXA-48_ in 73.2% of the carbapenem-resistant isolates [30].

Currently, only 3.5% of *Enterobacterales* and 4.5% of carbapenem-resistant *Enterobacteriaceae* are resistant to tigecycline in China, according to a survey by the China Antimicrobial Surveillance Network, and 3% (3 out of 100) prevalence of tigecycline resistance in Lebanon, but a previous study from our centre indicates that the phenotypic resistance rate of tigecycline was 7.85%(413 out of 5,258) in carbapenem-resistant Gram-negative isolates [24,31, 32]. While in the current study, 20.4% (31 out of 152) *K. pneumoniae* isolated from admitted patients were phenotypically resistant to tigecycline among carbapenem resistance. Among 31 isolates, the *tet(A)* gene was found to be present in four isolates, and one harboured *tet(A)*, as well as *tet(B)*. We found that all patients infected with *K. pneumoniae* isolates containing the *tet(A)* gene succumbed to their disease with outcome of respiratory tract infections and septic shock.

Conjugation experiments were done in two isolates out of four *K. pneumoniae* isolates that contain *tet(A)* gene on plasmid, as well as chromosome. Conjugative plasmids were non-transmissible in our isolates, but a current study in China indicates that the *tet(A)* gene in tigecycline resistance among CRKP isolates was present on transferable plasmid [10]. Conjugation experiments in the above *K. pneumoniae* isolates showed successful transfer of *bla*_NDM_ (2/2) and *bla*_OXA48_ (1/3) in *E. coli* J53 recipient out of two and three *bla*_NDM_- and *bla*_OXA48_-positive isolates. Several studies from China and the USA have reported the coexistence of *tet* genes with *bla*_NDM_ in Gram-negative bacteria, raising concerns about their potential to disseminate as drug-resistant strains [1,33, 34].

## Conclusion

Our study highlights the molecular characteristics and transmission dynamics of tetracycline resistance genes among TRKP and CRKP isolates in a tertiary care hospital of India. The *tet(A)* gene emerged as the predominant tetracycline resistance determinant in *K. pneumoniae* clinical isolates compared to the *tet(B)* gene. Although carbapenemase genes (*bla*_NDM_, *bla*_OXA-48_) were efficiently transferred via conjugation, tetracycline resistance genes were non-transferable, indicating their localization on non-conjugative plasmids or chromosomal elements. These findings suggest that carbapenem resistance poses a high risk for horizontal gene transfer, while the dissemination potential of tetracycline resistance genes remains limited in this setting.

## Limitations of the study

One limitation of this study was the lack of whole-genome sequencing data, which prevented a deeper understanding of the genetic makeup of carbapenem and tetracycline resistance genes in *K. pneumoniae*. Specifically, it determined the exact location of these genes in the genome or identified important nearby elements like insertion sequences, transposons and integrons that may help in their spread from one bacterium to another.

## Supplementary material

10.1099/acmi.0.001017.v4 Uncited Supplementary Material 1.

## Abbreviations

CLS: Clinical and Laboratory Standards Institute
CRKP: carbapenem-resistant *Klebsiella pneumoniae*
ET: endotracheal
EUCAST: European Committee Antimicrobial Susceptibility Testing
H₂S: hydrogen sulphide
MR: methyl red
PDA: phenylalanine deaminase
SCF: cefoperazone-sulbactam
TRKP: tigecycline-resistant *Klebsiella pneumoniae*
TRKP: tigecycline-resistant *Klebsiella pneumoniae*
TT: transtracheal
VP: Voges-Proskauer
