# Supplementary material for: Molecular characterization and transmission pattern of tetracycline resistance determinants in tigecycline and carbapenem resistant Klebsiella pneumoniae isolates at a tertiary care hospital in India
Source: Access Microbiol. 2026 Mar 12;8(3):001017.v4. doi: 10.1099/acmi.0.001017.v4 (PMC12982153; doi:10.1099/acmi.0.001017.v4)
Supplement: Uncited Supplementary Material 1. [file acmi-8-01017-s001.pdf]

Supplementary file:

Table S.1: PCR primer distribution in pairs used in the study

Resistance gene	PCR primer sequence 5'-3'	Amplicon size	Reference
bla$_{NDM}$	CACCTCATGTTTGAATTCGCC<br>CTCTGTCACATTCGAAATCGC	984	[20]
bla$_{OXA-48}$	TATATTGCATTAAGCAAGGG<br>CACACAAATACGCGCTAACC	848	
bla$_{KPC}$	TGTCACTGTATCGCCGTC<br>GTCAGTGCTCTACAGAAAACC	1011	
tet(A)	GCT ACA TCC TGC TTG CCT TC<br>CAT AGA TCG CCG TGA AGA GG	210	[18]
tet(B)	TTG GTT AGG GGC AAG TTT TG<br>GTA ATG GGC CAA TAA CAC CG	659	
tet(K)	TCG ATA GGA ACA GCA GTA<br>CAG CAG ATC CTA CTC CTT	169	
tet(M)	GTG GAC AAA GGT ACA ACG AG<br>CGG TAA AGT TCG TCA CAC AC	406	
tet(S)	CAT AGA CAA GCC GTT GAC C<br>ATG TTT TTG GAA CGC CAG AG	667	

Table S.2 Demographic data of patients infected with tigecycline and carbapenem resistant *K. pneumoniae* isolates and distribution of carbapenemase and tetracycline resistance gene in these isolates

P= Positive, M= Male, F= Female, IP= In-patient (Admitted)

Patient ID	Organism ID	blaNDM	blaOXA	blaKPC	*tet(A)*	*tet(B)*	*tet(K)*	*tet (M)*	*tet(S)*	Wards	Age	*Gender*	*Specimen*	*Patient Status*

**P1**	Kp1	P	P	P		CCM	40	M	Pus	IP
**P2**	Kp2		P			CCM	52	M	TT aspirate	IP
**P3**	Kp3					Surgical Gastroenterology	28	M	Drain Fluid (Pus)	IP
**P4**	Kp4	P	P	P		Surgical Gastroenterology	43	M	Intraop necrosum (Pus)	IP
**P5**	Kp5	P	P			CCM	21	M	Drain Fluid (Pus)	IP
**P6**	Kp6	P				Surgical Gastroenterology	37	M	Intra op bile (Pus)	IP
**P7**	Kp7		P			Surgical Gastroenterology	37	M	Intra op bile (Pus)	IP
**P8**	Kp8		P			Anaesthesia	46	F	CVP Line (Pus)	IP
**P9**	Kp9		P			Surgical Gastroenterology	28	M	Drain Fluid (Pus)	IP
**P10**	Kp10	P	P			Surgical Gastroenterology	37	M	Intra op bile (Pus)	IP
**P11**	Kp11					Anaesthesia	27	M	pancreatic necrosum (Pus)	IP
**P12**	Kp12		P			Nephrology	43	F	CVP Line (Pus)	IP
**P13**	Kp13	P	P			Surgical Gastroenterology	60	F	Drain Fluid (Pus)	IP
**P14**	Kp14	P	P			Anaesthesia	52	M	ET aspirate	IP
**P15**	Kp15					Surgical Gastroenterology	34	F	Bile (Pus)	IP
**P16**	Kp16					Apex Trauma Center	17	M	Pus	IP
**P17**	Kp17		P			Nephrology	52	M	Pus	IP
**P18**	Kp18					CCM	52	M	TT aspirate	IP
**P19**	Kp19					Apex Trauma center	50	M	Pus	IP
**P20**	Kp20		P	P	P	CCM	25	M	ET aspirate	IP
**P21**	Kp21					CCM	16	M	TT aspirate	IP
**P22**	Kp22					Apex Trauma Center	37	M	TT aspirate	IP
**P23**	Kp23					Emergency Medicine	66	M	Sputum	IP
**P24**	Kp24					CCM	16	M	TT aspirate	IP
**P25**	kp25	P	P			CCM	19	M	TT aspirate	IP
**P26**	Kp26					Neurosurgery	54	M	TT aspirate	IP
**P27**	Kp27		P			CCM	30	M	TT aspirate	IP
**P28**	Kp28	P				Neurosurgery	54	M	TT aspirate	IP
**P29**	Kp29					CCM	58	F	Pus	IP
**P30**	Kp30			P		CCM	53	M	TT aspirate	IP
**P31**	Kp31		P			Nephrology	52	M	Sputum	IP
